# Barriers and facilitators to access mental health services among refugee women in high-income countries: study protocol for a systematic review

**DOI:** 10.1186/s13643-020-01446-y

**Published:** 2020-08-16

**Authors:** Sarah DeSa, Akalewold T. Gebremeskel, Sanni Yaya

**Affiliations:** 1 Interdisciplinary School of Health Sciences, University of Ottawa, Ottawa, Ontario Canada; 2 Faculty of Health Sciences, University of Ottawa, Ottawa, Ontario Canada; 3 School of International Development and Global Studies, University of Ottawa, Ottawa, Ontario Canada; 4 The George Institute for Global Health, Imperial College London, London, UK

## Abstract

**Background:**

According to the United Nation High Commissioner for Refugee Global Trends report in 2019, on average, there are 2.7 refugees per 1000 national population in high-income countries, where girls and women attributed to 48% of the refugee population. Evidence shows high prevalence of mental health disorder among women refugees in comparison to the general population. To our knowledge, no systematic reviews have addressed access to mental health services for refugee women. The aim of this study will be to examine existing barriers and facilitators to accessing mental health services for refugee women in leading high-income countries for refugee resettlement.

**Methods:**

We designed and registered a study protocol for a systematic review. We will conduct a literature search (from inception onwards) in MEDLINE, EMBASE, PsycINFO, and CINAHL. Research articles having a qualitative component (i.e., qualitative, mixed, or multi-method) will be eligible. Study populations of interest will be refugee women at any age that can receive mental health services in leading high-income countries for refugee resettlement (e.g., 14 countries from North America, Europe, and Oceania). Eligibility will be restricted to studies published in English. The primary outcome will be all barriers and facilitators related to accessing mental health services. Two reviewers will independently screen all citations, full-text articles, and abstract data. Potential conflicts will be resolved through discussion. The study methodological quality (or bias) will be appraised using appropriate tools. Reporting will follow the Enhancing Transparency in Reporting the Synthesis of Qualitative Research (ENTREQ) statement. A narrative synthesis will be conducted, and summary of findings tables will be produced. As it will be a systematic review, without human participants’ involvement, there will be no requirement for ethical approval.

**Discussion:**

The systematic review will present key evidence on barriers and facilitators to access mental health services among refugee women in leading resettlement countries. The findings will be used to inform program developers, policymakers, and other stakeholders to enhance mental health services for refugee women. The final manuscript will be disseminated through a peer-reviewed journal and scientific conferences.

**Systematic review registration:**

PROSPERO CRD42020180369.

## Background

According to the United Nation High Commissioner for Refugee (UNHCR) figures, as of May 2019, there were 70.8 million people forcibly displaced globally [1]. The experience of forcible migration has been documented as a complicated and stressful life event that can take a toll on mental health [2, 3]. In addition to facing changes in their surroundings, refugee populations are often neglected when it comes to adequacy of health services, including crucial gaps in access and delivery of mental health services [4]. According to the World Health Organization (WHO), mental health is a state of well-being in which an individual realizes his or her own abilities, can cope with the normal stresses of life, can work productively, and is able to contribute to his or her community [4].

On the global front, in 2018, the WHO set in place a technical guidance report for mental health promotion and mental health care plan for refugees and migrants under their policy framework for 2020 [5]. This framework addressed ways to promote refugee’s social integration, address and overcome barriers to accessing mental health care, facilitating engagement and utilization of services [5, 6].

Approximately one third of people who have obtained refugee status live in high-income countries [7]. The number of refugees and asylum seeker has been showing significant increase among major host and high-income resettlement countries [8, 9]. Global departures grew in 2019, with 63,726 refugees departing for resettlement [10]. The USA remains the country with the highest number of arrivals with 21,159 persons arriving in 2019, an almost 24% increase from 2018. Canada received 9031 arrivals, a 17% increase from 2018, followed by the UK with 5774 arrivals (just over a 1% increase from the previous year). Other countries that have been on the UNHCR’s top 10 resettlement countries list in the past 10 years were Sweden with 4993 refugees arriving in 2019, Germany (4622), France (4544), Australia (3464), Norway (2351), Netherlands (1857), and Switzerland (990) [10]. With the increase of refugees in leading resettlement countries, it is important to comprehend the circumstances in accessing the mental health care systems and interventions to deal with those challenges [8, 9].

Refugee acceptance, health service availability, and accessibility contexts are different among resettlement countries [8, 11]. The variation in cultural, political, economic, and social frameworks can play a large role in how a country prioritizes mental health service allocation for refugee populations [11]. Mental health care policies and entitlements for refugee and asylum seekers also differ in each host country [9, 12]. Refugee mental health care services in most resettlement countries in high-income countries can consist of lengthy treatments, delivered by scarce and expensive mental health professionals [12]. There are system-wide obstacles hindering refugees and asylum seekers to accessing mental health services [12]. The health policy of majority of high-income and UNHCR refugee settlers ensures the access to health care. However, for refugees at country level, there are many associated obstacles to fully access health services including mental health services [12, 13]. Furthermore, there is evidence that shows restrictive entry and integration policies are linked to poor migrant health outcomes in high-income countries [13]. It is worthwhile to discuss how these countries of high refugee resettlement are affected by migration policy to assess its critical effect on access to mental health services.

Studies have documented similar patterns and risk factors that are specific to female refugees compared to their male counter parts [14]. For example, the health needs of Syrian refugee women who have migrated to a resettlement country like Canada, although residing in a metropolis like Toronto, reported to have unmet health needs. These health needs included ineffective access to health services and mental health services linked to the disposition of gender-based violence and trauma [15–17]. Furthermore, studies have shown to a concern for psychological risks among refugees related to war, violence, and trauma protection needs among women and girl refugees, and challenges in managing mental health disorders [8, 18, 19]. Such psychological risk plays its intrinsic role in the eligibility of resettling in another country, including fitting a criteria such as having survived torture or serious violence, being a woman or girl at risk of abuse and exploitation, or facing persecution because of gender or sexual orientation, among many other devastating scenarios [10].

In general, identifying and addressing barriers and facilitators that directly impact needs, access, and use of mental health services can be worth exploring in order to provide better care and improved circumstances for vulnerable populations such as refugee women. When it comes to identifying barriers to mental health access and utilization, studies have illustrated the need for improving social and structural constructs to ease the use of mental health services among refugee population [9, 20, 21]. Barriers that have been identified to influence refugee mental health include understanding a new health system, structural barriers such scheduling or restrictive timing, linguistic barriers, attitudes, and perceived discrimination [20–22].

Another common problem faced within these resettlement countries is that neither refugees themselves nor their clinicians are fully aware of the exact entitlements of mental health services for refugees [12]. Additionally, systemic barriers, the social determinants of health like exposure to social exclusion, stigmatization, discrimination, and low social status, also have a negative effect on accessing mental health services among refugees in major resettlement countries [23–25]. While there are many determinants that affect access to mental health services in high-income settings, there is a recognized need for services to be accessible, acceptable, and effective within resettlement countries, and should correspond with the needs and difficulties of refugees.

While there are lots of studies that examine the barriers to accessing mental health services, there are limited studies that identify facilitators to accessing mental health care among the refugee women population. Few studies have identified some facilitators in accessing mental health services among refugees, including provision of culturally sensitive intervention [25] and provision of gender concordant services [14, 16, 26].

Despite a growing body of literature, which examines barriers that influence mental health, there is a lack of systematic reviews that specifically examine the evidence on barriers and facilitators to access mental health services for women refugees and asylum seekers in high-income countries [8, 27]. In this context, the aim of this study will be to examine existing barriers and facilitators to accessing mental health services for refugee women in leading high-income countries for refugee resettlement.

## Methods

### Protocol registration and reporting

The present protocol has been registered within the PROSPERO database (registration number CRD42020180369) and is being reported in accordance with the reporting guidance provided in the Preferred Reporting Items for Systematic Reviews and Meta-Analyses Protocols (PRISMA-P) statement [28] (see checklist in Additional file 1). This review will be conducted following the Cochrane Collaboration Handbook of Systematic Reviews [29]. The proposed systematic review will be reported in accordance with the reporting guidance provided in the Preferred Reporting Items for Systematic Reviews and Meta-analyses (PRISMA) statement [28] and the Enhancing Transparency in Reporting the Synthesis of Qualitative Research (ENTREQ) statement [30].

### Selection criteria

We have defined the following predefined eligibility criterion for the planned systematic review (see Additional file 2 for more details).

#### Study design

Eligible studies will be reports of original research, peer-reviewed articles having a qualitative component (i.e., qualitative, mixed, or multi-method studies).

#### Participants

We will include studies involving refugee women (regardless of age) that can receive mental health services. Studies conducted in both sexes will be considered for inclusion, but only data for women will be extracted.

#### Context

Eligible studies will involve one or more type of usual standard mental health service for refugee women, including abuse support, addiction support, counseling, crisis support, psychiatric and psychological assessments and treatments, and support groups.

#### Comparison or control group

There is no comparison group for this study.

#### Outcomes of interest

The primary outcome will be all barriers and facilitators related to accessing mental health services.

#### Setting

We will include studies conducted in leading high-income countries for refugee resettlement. Eligible countries will be selected based on data from 2009 to 2019 in the UNHCR’s global resettlement needs reports [10]. According to this data source, the world’s leading 14 resettlement countries for refugees within the last decade are as follows:

- North America: Canada and USA
- Europe: Belgium, Denmark, Finland, France, Germany, Netherlands, Norway, Switzerland, Sweden, and UK
- Oceania: Australia and New Zealand

### Exclusion criteria

We will exclude reviews, editorials, commentaries, conference abstracts, dissertations, and other gray literature. Additionally, this review will exclude non-English articles, studies conducted only in men, and studies reporting data from countries outside of the UNHCR’s leading resettlement countries from the past decade.

### Information sources and search methods

The primary source of literature will be a structured search of major electronic databases (from inception onwards): MEDLINE (Ovid), EMBASE, PsycINFO, and CINAHL. The search strategies will comprise the following stages. First, a search of MEDLINE (Ovid) to identify relevant keywords contained in the title, abstract, and subject descriptors. Second, we will identify the synonyms and related terms for searches in EMBASE, PsycINFO, and CINAHL. In addition, we will perform hand-searching of the reference lists of included studies, relevant reviews, or other relevant documents. Content experts and authors who are prolific in the field will be contacted. The search will include a broad range of MeSH terms and keywords related to mental health services, accessibility, refugee, asylum seeker, women/female, and qualitative research. A draft search strategy within multiple databases is provided in Additional file 3. MeSH terms related to mental illness were not included, as this review focuses on accessing mental health services and not necessarily the presence of mental illness.

### Selection of studies

Citations will be imported into the Zotero citation management software and uploaded in a zip file. The articles retrieved from searches in each database will be uploaded into the Covidence article management system to be screened by two authors within the Covidence database for their relevance and eligibility to the review. This will include title and abstract screening, followed by full-text screening against the eligibility criteria for studies deemed potentially eligible. Disagreements will be settled through discussion. The PRISMA (Preferred Reporting Items for Systematic Review and Meta-Analyses) flowchart will be used to document the selection process [28].

### Data extraction and management

Following full-text screening, data will be independently extracted from the retrieved eligible studies by two of the reviewers (ATG and SD). Disagreements will be settled through discussion with a third reviewer (SY). The authors will adapt a data collection form based on the needs of the review from a standardized data extraction form by the Cochrane Handbook [29]. The data extracted will include all details specific to the review question, fulfilling the requirements for a narrative synthesis. This includes the following information from each article: (i) authors and publication year, study setting, and study aim or hypothesis; (ii) sample characteristics, design and data collection methods, and outcome measures; and (iii) study findings. We will also contact primary study authors for key information when data are ambiguous or missing from the included studies.

### Assessment of risk of bias in included studies

A critical appraisal of included studies will be conducted by two reviewers independently. All disagreements will be resolved through discussion or consultation with a third reviewer as needed. Results from the appraisal will be summarized narratively to highlight strengths and limitations within and across studies. Tables or figures will be used to present and/or graphically summarize results.

The reviewers will evaluate the studies using the appropriate Critical Appraisal Skills Programme (CASP) checklists [31]. Included studies will be assigned an overall score of “high” (9–10), “moderate” (7.5–9), or “low” (less than 7.5) overall quality. Studies will not be excluded or weighted based on the quality of the reporting assessment. The results of the appraisal will instead be used to inform data interpretation and help confirm the validity of review findings and conclusions.

### Certainty of evidence

The GRADE-CERQual (“Confidence in the Evidence from Reviews of Qualitative research”) approach will be applied to assess and summarize confidence in key findings [32]. This will provide overall confidence in each of the key findings. Two reviewers will independently assess certainty of the evidence using the GRADE-CERQual approach [32]. Disagreements will be resolved through discussion. Results will be presented in GRADE-CERQual summary of qualitative findings tables [32].

### Data synthesis

Evidence tables of an overall description of the studies, including data from each paper that provided details of study characteristics, context, participant age and sex, outcomes, and conclusion. A narrative synthesis will be conducted, a method that is ideal for synthesizing evidence from a wide range of research questions and study designs with qualitative, mixed, or multi-method approaches, as the emphasis is on an interpretive synthesis of the narrative findings of research [33]. Synthesis of data will be described in a narrative synthesis, grouped by study type and participant characteristics and review objective and outcome. Accordingly, barriers and facilitators of mental health services for refugee women in high-income countries will aim to inform policy to improve access to mental health services for refugee women.

## Discussion

Mental health care policies and entitlements differ in each country [8, 12], and across the leading resettlement, high-income countries, there is a variance in how the types of mental health services are available and accessed for refugee populations [8]. For example, it has been estimated that in Germany, in 2015, only 5% of refugees in need of mental health care received treatment [12]. On the other hand, in 2016, the Mental Health Commissioner of Canada released information on the provincial needs to address and reduce disparities and improve access to mental health services in diverse communities [4]. Similar reports sought to improve access and outcomes of mental health services for immigrants, refugees, and racialized ethno-cultural groups [3, 4].

This systematic review will summarize the evidence regarding mental health care barrier and facilitator study characteristics, context, and participant’s sex and age, to address the experiences faced by refugee women when accessing mental health services in leading resettlement countries. The findings of this research may be applied to enhance existing mental health service access for refugee women in leading resettlement countries.

This systematic review and its evidence synthesis will be published in a peer-reviewed journal and presented at different conferences and scientific meetings. This research aims to ultimately inform policymakers and stakeholders in mental health service promotion. Additionally, this review hopes to contribute to the campaign for effective delivery of ample mental health service resources for refugee women, such that follow the high standards set in high-income, leading resettlement countries. This review will provide insight on the extent to which health system, specifically mental health care, in resettlement countries enables or challenges refugee women to accessing those services.

This protocol outlines the methodological process of a systematic review that will gather qualitative data in order to examine existing barriers and facilitators to accessing mental health services for refugee women in leading resettlement countries. There are several limitations of our planned systematic review methods. There is an exclusion of research published in languages other than English, which can result in the exclusion of valuable data. Additionally, some data may be unrepresented, underreported, or misreported due to sensitive and highly stigmatize nature of mental health issues among refugee populations. This may result in publication bias and methodological quality issues.

## Supplementary information

**Additional file 1:.** PRISMA Checklist.

**Additional file 2:.** Study selection criteria.

**Additional file 3:.** Search terms.

## Abbreviations

CASP: Critical Appraisal Skills Programme
PRISMA-P: Preferred Reporting Items for Systematic Review and Meta-analysis Protocols
PROSPERO: International Prospective Register of Systematic Reviews
UNHRC: United Nation High Commissioner for Refugee
WHO: World Health Organization
