# Supplementary material for: Barriers and facilitators to access mental health services among refugee women in high-income countries: study protocol for a systematic review
Source: Syst Rev. 2020 Aug 16;9:186. doi: 10.1186/s13643-020-01446-y (PMC7429857; doi:10.1186/s13643-020-01446-y)
Supplement: Supplementary file 3 — Additional file 3:. Search terms. [file 13643_2020_1446_MOESM3_ESM.docx]

**Search Terms: Medline (OVID)**

**Set 1: Intervention**

*A) MeSH*

Mental Health Services/ or Health Services Accessibility/ or Ethnopsychology/ or psychiatry/ or psychology/ or psychopharmacology/ or exp community mental health services/ or exp counseling/ or exp emergency services, psychiatric/ or exp psychiatric somatic therapies/ or exp psychological techniques/ or exp psychotherapy/

*B) Keyword*

Mental adj2 health adj2 (service* or Program*) or ethnopsych*

**Set 2: Population**

*A) MeSH*

Refugees/ or Asylum seekers/

*B) Keyword*

Refugee* or (asylum* adj2 seeker*) or (displac* adj2 person*) or (forced adj2 migrant*) or (undocumented or unauthorized or illegal)) adj2 (immigrant* or alien* or worker*)

**Set 3: Search Alone**

*A) MeSH*

Women/ or Female/

*B) Keyword*

Wom?n or girl* or female*

**Set 4: Study Design**

Qualitative Research/

**Search Summary:** Set 1 (A or B) and Set 2 (A or B) and Set 3 (A or B) and Set 4
